# Microlearning beyond boundaries: A systematic review and a novel framework for improving learning outcomes

**DOI:** 10.1016/j.heliyon.2024.e41413

**Published:** 2024-12-20

**Authors:** Wali Khan Monib, Atika Qazi, Rosyzie Anna Apong

**Affiliations:** a Centre for Lifelong Learning, Universiti Brunei Darussalam, Gadong BE 1410, Brunei Darussalam; b School of Education, Taylor's University Lakeside Campus, Subang Jaya 47500, Malaysia; c School of Digital Science, Universiti Brunei Darussalam, Gadong BE1410, Brunei Darussalam

**Keywords:** Microlearning, Learning outcomes, Conceptual framework, Higher education, And corporate sector

## Abstract

Microlearning has become increasingly popular not only in education sector but also in corporate sector in recent years. However, its definition and didactics conceptualization, integration into instruction design, and effects on learning outcomes remain largely underexplored in terms of synthesized findings. Consequently, challenges persist in clarifying microlearning definition, and didactics, and designing effective microlearning instruction to yield improved learning outcomes. To address these gaps, we analyzed 40 relevant studies following PRISMA guidelines. Based on the findings, we maintain that microlearning is an instructional approach that delivers targeted, action-oriented, bite-sized content to achieve specific objectives within a short period, typically within a few seconds or minutes. The most important findings from the reviewed studies indicate that microlearning has positive impact on learning outcomes. The key learning outcomes identified, categorized according to Bloom's Taxonomy, include cognitive (knowledge acquisition, retention, improvement, recall, transfer, and application as well as critical thinking, problem-solving, professional, feedback and self-regulation skills, core competencies such as digital and pedagogical competence, and performance like test performance), behavioural (presentation skills development, task or work performance, higher completion rates, behavioural patterns, engagement, and collaboration), and affective (positive perceptions and attitudes, increased motivation, satisfaction, and improved self-efficacy). Based on Andragogy in Practice Model, Bloom's Taxonomy, and Sweller's CLT, we proposed a novel microlearning instructional design framework consisting of three integral components—differences consisting of individual, situational, and subject differences; guiding principles including specific-objective, bite-sized content, appropriate timeframe, interactive and engaging content, personalization, and selecting appropriate delivery medium and mode; and learning outcomes (cognitive, behavioural and affective). The proposed framework aims to integrate microlearning into instruction to improve learning outcomes. Educators, instruction designers, and policymakers can use the framework to design microlearning instruction to improve learning outcomes.

## 1 Introduction

The 21st century has accelerated the shift from traditional to computerized, learner-centred education, with technology remaining a cornerstone for future generations [1]. The increasing flexibility and affordability of mobile devices have further democratized education, making learning systems more accessible to both learners and educators worldwide [2]. Smart technology applications have become pivotal in sustaining learning [3], and the learners' busy lifestyles have shifted their information-seeking behaviours, with many preferring small, digestible chunks of information delivered quickly. Microlearning as an instructional approach [4] to fulfilling such preferences is one of the best options. According to Leong et al. [5], people are used to obtaining information at their fingertips and finding answers in just a few minutes. With the information presented in small manageable segments [6], microlearning makes knowledge transformation less arbitrary and more practical [7]. However, a largely agreed definition does not yet exist due to the fact that microlearning is still evolving and is in the early stage [8], particularly when it comes to the extent of the duration of instruction. Literature in this regard is scattered, with a large gap in the duration proposed for the delivery of microcontent. Hug [9] states that microlearning lacks a precise definition and derives from diverse interpretations across dimensions like time, content, curriculum, form, process, mediality, and learning type. The term ‘microlearning’ by itself is not agreed upon among the users and has been referred to by different nomenclatures and with various didactics as to whether it is an approach, theory, strategy, instructional method or mode. This reflects the diversity in how the term is understood and applied. It tends to be used to refer to small, digestible chunks of information focused on a single learning objective that is need-based, action-oriented, and compatible with smart e-learning tools.

With regard to content design, microlearning has several key characteristics that prove to be effective in managing cognitive load, including intrinsic, extrinsic, and germane cognitive loads. CLT which defines cognitive load as the amount of information working memory can hold at once, underpins the principles of microlearning [10]. To ensure optimal learning outcomes, microlearning content is designed to be bite-sized, multisensory, interactive, personalized, and self-contained. The diverse and valuable learning resources should be accessible to learners’ at their convenience, either independently or as part of a curriculum, all tailored to specific learning needs.

To improve the effectiveness of the designed content, it is essential to choose the appropriate mediality for delivery. Microlearning content can be presented in various audio-visual formats, such as videos, audio, and images [11]. Short videos, in particular, have been identified as highly effective in microlearning [12], and the use of video clips and short animations has been emphasized [13]. In this context, mobile devices serve as a convenient tool for microlearning activities, allowing learners to access content anytime and anywhere [14]. While some critics raise concerns about its ability to effectively deliver complex topics , its benefits in managing cognitive load remain significant in learning contexts. Other crucial benefits of microlearning and relevant tools include improved learner engagement [15], flexibility [16,17], accessibility [18,19], focused content [20], device friendliness [21], short duration [22], independence and responsiveness [23] and to name a few. These and other hallmark characteristics have led to increased adoption and research interest in microlearning. In recent years, the volume of publications on microlearning has substantially risen with evaluating its effectiveness [11] and design [24] as the most explored areas.

Despite the growing interest in microlearning and numerous studies on its effectiveness, the results are scattered and mixed [4]. This indicates the need for a comprehensive review of the available research to systematically synthesize knowledge and determine its effectiveness in improving learning outcomes [25]. While there are reviews on microlearning, they vary significantly from the current study in the methodology (e.g., [[5], [8], [11]]) and scope (e.g., [26]). For example, Taylor and Hung [8] conducted a scoping review on the effects of microlearning, Sankaranarayanan et al. [11] performed a bibliometric analysis on microlearning in diverse contexts, and Leong et al. [5] carried out a review of the trends in microlearning. In terms of scope, Wang et al. [26] limited their study scope to the efficacy of microlearning in improving self-care capability. Zhang and West [27] emphasize the importance of exploring both quantitative and qualitative evidence to gauge the impact of microlearning. It is further stated that an exploration of its efficacy in delivering intangible knowledge becomes particulalry crucial in this endeavour.

By synthesizing the existing research on microlearning, this review aims to provide a comprehensive understanding of the effectiveness of microlearning and inform the development of best practices for designing and implementing microlearning instructions. While past reviews have often concentrated on specific aspects of microlearning—such as its effectiveness in particular contexts e.g., the effects of microlearning on EFL students’ English speaking [25]—the current systematic review expands its scope to provide a comprehensive synthesis of microlearning outcomes across diverse settings. We included quantitative, qualitative, and mixed-method studies, offering deeper insights into microlearning effectiveness across varied settings while addressing gaps in previous reviews. This review aimed at finding answers to the following research questions (see Table 1).

**Table 1:** Research questions, focus, and description.

Research Questions	Focus	Description
**RQ1.** How is the definition of microlearning and its didactic conceptualized in the selected studies?	Understanding the definition and didactics	**RQ1** aims to explore the definition, nomenclature, and didactics of microlearning in the selected literature.
**RQ2.** What are the effects of microlearning on learning outcomes?	Impact on learning outcomes	**RQ2** aims to explore the educational effects of microlearning on learning outcomes in various fields, categorizing these outcomes based on Bloom's Taxonomy. It also explores how to design effective microlearning to improve learning outcomes.

## 2 Theoretical framework

CLT, introduced by John Sweller in the late 1980s, explains the concept of cognitive load, which represents the load on the cognitive system during task performance. The term ‘cognitive load’ refers to the mental activity that occurs concurrently within working memory [28]. CLT comprises task-based (*mental load*) and learner-based (*mental effort*) dimensions, which collectively impact the *performance* of individuals [29] and the three dimensions including mental load, mental effort, and performance can measure cognitive load [30]. *Mental load* pertains to the demands imposed by the task itself, including task-intrinsic aspects related to elements interactivity, and task-extraneous aspects related to instructional design. When the intrinsic cognitive load caused by the inherent complexity of the information and the extraneous cognitive load caused by instructional design are high, it can be detrimental to learning [29,31]. This is because the working memory, which is responsible for processing information, may become overwhelmed and unable to handle the cognitive demands as the cognitive load exceeds the capacity of the working memory to effectively process and retain information [29]. They further argue that the extraneous cognitive load is a result of the instructional format rather than being an inherent characteristic of the materials.

*Mental effort*, on the other hand, represents the cognitive capacity and resources allocated to address the task demands [30]. If learners perceive their efforts as unproductive to success, they may not be motivated to put in the necessary mental effort [32]. The mental effort level involves task or environmental characteristics, subject characteristics, and interactions between both [30]. They further state that complex cognitive tasks that impose a high mental load typically require a high level of mental effort.

*Performance* reflects the outcomes achieved by the learner as a result of their engagement with the task. Performance-related load is called germane cognitive load which refers to the cognitive effort required for meaningful learning and schema construction. According to Sweller et al. [29], when the total cognitive load is manageable, educators have the opportunity to guide learners' attention towards processes that are important for learning and constructing mental frameworks (increasing germane cognitive load). At the same time, educators can redirect learners' attention away from processes that are not directly related to learning (reducing extraneous cognitive load). By employing appropriate instructional designs, educators can help reduce unnecessary cognitive load and focus learners’ attention on cognitive processes that contribute directly to building an understanding of the subject matter and mental schemas. The theory suggests that learning happens best under conditions that align with cognitive architecture [32]. Furthermore, learners have limited cognitive resources, and it is important to use these resources effectively to achieve optimal learning outcomes. Drawing from the above discussion, the present study was firmly grounded in CLT.

In addition to the CLT, two other models are also used, including Andragogy in Practice Model [33] and Bloom's Taxonomy [34]. The Andragogy in Practice Model comprises three dimensions: (1) learning goals and purposes, (2) individual and situational differences, and (3) core principles of adult learning. Andragogy emphasizes individual learning, which contributes to personal, institutional, and societal progress [33]. Demographic variables are derived based on the second dimension of Andragogy, capturing individual, situational, and subject differences. Bloom's Taxonomy categorizes learning into three domains: cognitive (intellectual skills), affective (attitudes and values), and psychomotor (physical skills) [34], guiding the categorization of learning outcomes.

## 3 Methodology

This study followed the PRISMA (Preferred Reporting Items for Systematic Reviews and Meta-Analyses) guidelines, ensuring a comprehensive analysis and synthesis of the existing literature on the effectiveness of microlearning. With pre-defined eligibility criteria, we included a diverse range of studies, including qualitative, quantitative, and mixed-method empirical research for in-depth understanding. Quantitative studies provide measurable and statistically significant data, offering insights into the extent and patterns of the impact. On the other hand, qualitative studies capture in-depth perspectives, experiences, and contextual nuances that are often missed in purely numerical analyses. In addition, mixed-method studies were included to bridge these two approaches, integrating statistical data with rich descriptive insights to provide a holistic view. By incorporating all three methodologies, the study ensures covering both the breadth and depth of microlearning effectiveness across different learning contexts and populations. This comprehensive approach not only strengthens the validity of the findings but also offers a balanced understanding, accommodating different types of evidence and perspectives. We employed a quality appraisal strategy based on Dybå and Dingsøyr [35] criteria to assess the rigor and credibility of the included studies. Subsequently, we devised a coding scheme to systematically analyze the publications in the final selection. The learning outcomes were synthesized in accordance with Bloom's Taxonomy.

### 3.1 Search strategy

For a comprehensive literature review, we searched several reputable databases such as SAGE, Taylor & Francis, SpringerLink, IEEE Xplore, Emerald, Scopus, and ERIC. Our search focused on original articles published in various disciplines from 2020 onwards. We utilized specific keywords including microlearning, MiroLearning, micro-learning, just-in-time learning, bite-sized learning, nano-learning, OR mini-learning AND learning outcomes, performance, OR effectiveness. Furthermore, we incorporated synonymous terms such as small chunks, short training, short video training, segmented learning, mini-lessons, microcontent, microunits, microtasks, snippets, nuggets, and snack-sized learning for inclusiveness. The search resulted in an initial pool of 3613 publications.

### 3.2 Inclusion and exclusion criteria

Table 2 presents the inclusion and exclusion criteria for the selected studies. Only papers published between 2020 and 2024 were considered, ensuring that the analysis is based on the most current research. Studies were included if they investigated the effectiveness or intervention of microlearning, ensuring relevance to the research objectives. Only peer-reviewed articles with full-text accessibility and those published in English and used microlearning as the primary mode of instruction were selected to maintain consistency and prevent translation-related challenges. Additionally, studies comparing microlearning with other learning methods, such as online and face-to-face learning, were included. The review focused on original articles. Exclusions included conference papers, proceedings, and grey literature. Furthermore, studies outside the specified publication date range, not directly related to microlearning, inaccessible in full text, or published in languages other than English were excluded. This thorough selection process ensures that the included studies align with the research objectives and meet quality standards.

**Table 2:** Inclusion and exclusion criteria for selected studies.

Inclusion	Exclusion
Papers published between 2020 and 2024.	Papers not published from 2020 to 2024.
Papers investigated microlearning effectiveness or intervention.	Papers not related to microlearning effectiveness or intervention.
Papers that were accessible.	Papers whose full text was not accessible.
Papers that were written in the English language.	Papers published other than in English Language.
Original peer-reviewed articles.	Grey literature.

### 3.3 Study selection

**Fig. 1:**
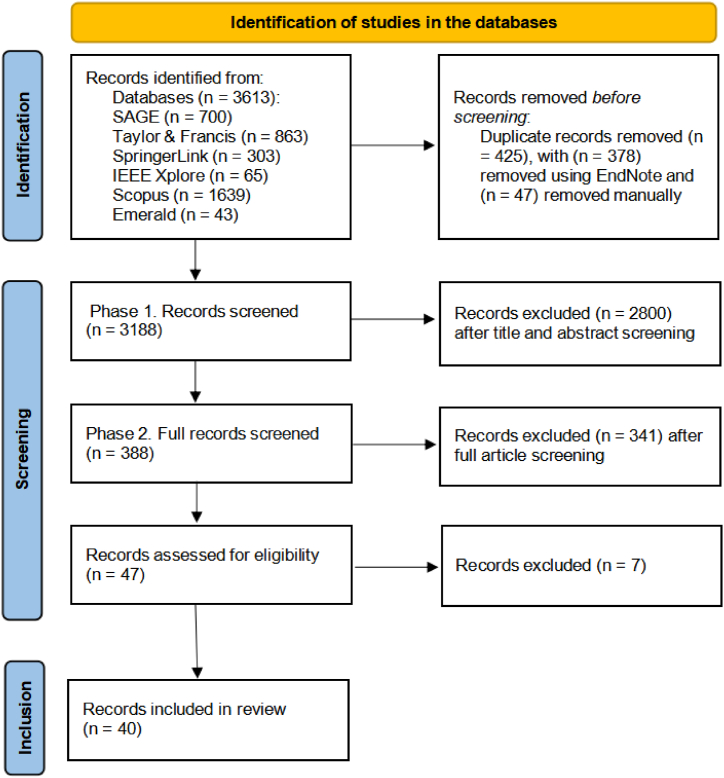
PRISMA flow diagram for study selection.

As depicted in Fig. 1, the initial search yielded 3613 articles from different databases, including SAGE (n = 700), Taylor & Francis (n = 863), SpringerLink (n = 303), IEEE Xplore (n = 65), Scopus (n = 1639), and Emerald (n = 43). Afterward, we utilized EndNote X9.0, a reference management software, to manage the references. Initially, duplicates were identified and removed (n = 378) using EndNote, with any remaining duplicates manually eliminated (n = 47). Next, we screened the titles and abstracts of the articles to determine their relevance for inclusion in the review, eliminating (n = 2800) articles and leaving us (n = 388) articles. Subsequently, we reviewed the full text of potentially relevant articles which led to (n = 341) being removed and (n = 47) articles left for quality assessment. After applying the quality appraisal, 40 articles were retained for analysis. Any disagreements during the screening process were resolved through consensus, with re-evaluation of the articles using the same criteria in our regular meetings.

### 3.4 Quality assessment criteria

We employed three primary criteria (rigor, credibility, and relevance) to evaluate the quality of the selected articles, as suggested by Dybå and Dingsøyr [35] (see Table 3). The quality evaluation used for each article focused on eight key aspects: (1) clarity in addressing the research problem, (2) explicit research aims, (3) adequacy of context description, (4) appropriateness of the research design, (5) rigor in methods (subjects, instruments, data collection, and analysis), (6) rigor in data analysis, (7) clarity of findings, and (8) overall value to research or practice. Two researchers independently assess each study to ensure objectivity, and any discrepancies are addressed through discussion to reach consensus in regular meetings. If inconsistencies or issues arise, the studies are reassessed until an agreement is achieved. Out of the 47 studies subjected to quality appraisal, only 40 articles met the criteria and were included for analysis.

**Table 3:** Quality assessment.

Quality criteria
1. Does the study clearly address the research problem? 2. Is there a clear statement of the aims of the research? 3. Is there an adequate description of the context in which the research was carried out? 4. Was the research design appropriate to address the aims of the research? 5. Does the study clearly determine the research methods (subjects, instruments, data collection, data analysis)? 6. Was the data analysis sufficiently rigorous? 7. Is there a clear statement of findings? 8. Is the study of value for research or practice?

### 3.5 Data extraction

A Microsoft Excel form was utilized to extract and record data from the selected articles. Information such as author(s) and year, country, field of study, research question(s) or objectives, method, population, sample size, research instrument, and findings were extracted (see Appendix A). Most of the studies were conducted in 2022, with a primary focus on medical and healthcare fields. Notably, a significant portion of the research originated from the USA. The predominant use of quantitative methods was evident, with a majority concentrating on learners and employing a combination of data collection tools.

Additionally, we developed a separate form to extract the findings (see Appendix B). The appendix consists of references, learning outcomes, terms, and didactics. The outcomes included knowledge, comprehension, skills, competence, performance, engagement, satisfaction, usefulness, motivation or interest, self-efficacy, confidence, anxiety and stress, attitude or perception, behaviour, relevance, and socialization. These learning outcomes were then categorized based on Bloom's Taxonomy. Each researcher independently extracted data from the included studies. Following the data extraction process, the datasets were compared and carefully examined to identify and resolve any potential discrepancies.

## 4 Results

In the following section, we present the results and address the research questions in detail.

### 4.1 RQ1. How is the definition of microlearning and its didactic conceptualized in the selected studies?

The analyzed studies present a range of conceptualizations of microlearning. Microlearning, despite various attempts to define it, lacks a universally accepted definition [36]. It is frequently mentioned with the concepts of eLearning, mobile learning, and informal learning [37]. Some view it as a form of eLearning [27], a successor to eLearning [38], or one of the eLearning methods or approaches that can help thwart a swing in the objectives of the learning, with one of its functions [22]. Examples of microlearning include a 3-min video demonstrating new software features, a 5-question quiz on grammar for quick reinforcement, or digital flashcards providing daily vocabulary, all designed to deliver actionable information and reinforce learning in manageable segments. Although there may be slight variations in the definitions, what this study revealed is that the core understanding of microlearning centres around delivering small, targeted units of learning content that are tailored to specific learning objectives.

**Fig. 2:**
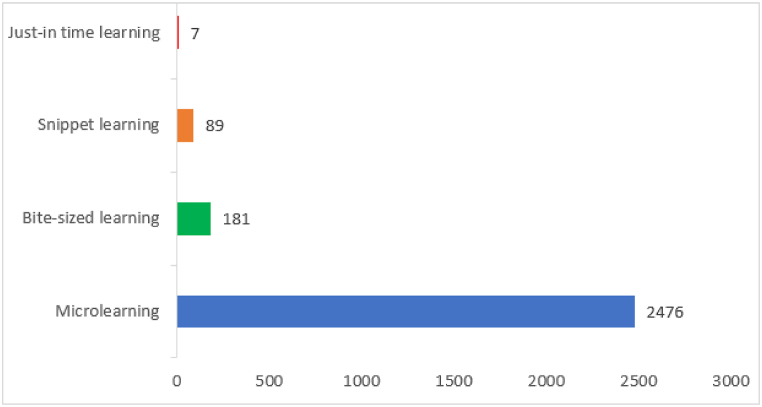
The frequency of the terms used for microlearning.

Various terms and didactics have been used to convey the concept of microlearning within the selected studies (see Appendix B). Out of the 40 studies analyzed, the term ‘microlearning’ and its alternatives, such as ‘micro-learning,’ ‘micro learning,’ and ‘MicroLearning,’ were frequently employed, with a combined total of (n = 2476) mentions (see, Fig. 2). Among them, ‘microlearning’ without a space between ‘micro’ and ‘learning’ was the most commonly used, appearing (n = 2247) times. The variant ‘micro-learning’ with a hyphen was mentioned (n = 204) times, while ‘micro learning’ with space was used (n = 25) times. In addition to ‘microlearning,’ alternative nomenclature was also identified in the studies. The nomenclature ‘bite-sized learning’ was used (n = 181) times, highlighting the notion of delivering content in small, manageable units. ‘Snippet learning’ was mentioned (n = 89) times, indicating the use of brief and focused learning experiences. ‘Just-in-time learning’ was referenced (n = 7) times, emphasizing the importance of providing learners with timely and relevant information. The term ‘bite-size’ was selectively included when referring to learning as ‘bite-sized learning,’ but it was not included when discussing other contexts like ‘bite-sized content’ or ‘bite-sized chunks.’

In some studies, notable variations were observed in the usage of ‘bite-sized learning.’ For example, Naseem et al. [39] consistently used the term ‘bite-sized learning’ 28 times, highlighting their focus on this specific term. On the other hand, in some studies, such as that of Ho et al. [17], the term ‘micro-learning’ appeared 10 times while ‘bite-sized learning’ was used 136 times, indicating interchangeability between these terms within their research. Manning et al. [40] employed various terminologies including ‘Microlearning’ 2 times, ‘micro learning’ 1 time, ‘bite-sized learning’ 19 times, and ‘bite-sized teaching’ 20 times. The findings underscore the extensive usage of the term ‘microlearning’ and its variants, reflecting its widespread recognition and adoption in the field. The alternative nomenclatures, such as ‘bite-sized learning,’ ‘snippet learning,’ and ‘just-in-time learning,’ also contribute to the understanding of microlearning.

Regarding the didactics of microlearning, 26 studies used the didactics ‘approach’: 13 studies used it alone, while 13 combined it with other didactics such as instructional method, methodology, technique, tool, pedagogy, strategy, design, format, or model. Nine studies did not use the didactic approach but instead employed other didactic such as learning modality, program, module, technology-improved learning, method, format, and/or strategy. Notably, five studies did not explicitly mention the didactic aspect. The consensus among these studies was that microlearning involves the delivery of bite-sized content or learning experiences that are tailored to meet specific learning needs. Different synonymous words, such as nuggets, bite-sized, chunks, segmented, or small units, were utilized to convey the idea of shortness and focused learning experiences. The current study revealed that microlearning is an instructional approach that delivers targeted, bite-sized content to diverse learners, typically lasting from a few seconds to a few minutes, each designed to achieve a specific objective.

### 4.2 RQ2. What are the effects of microlearning on learning outcomes?

**Fig. 3:**
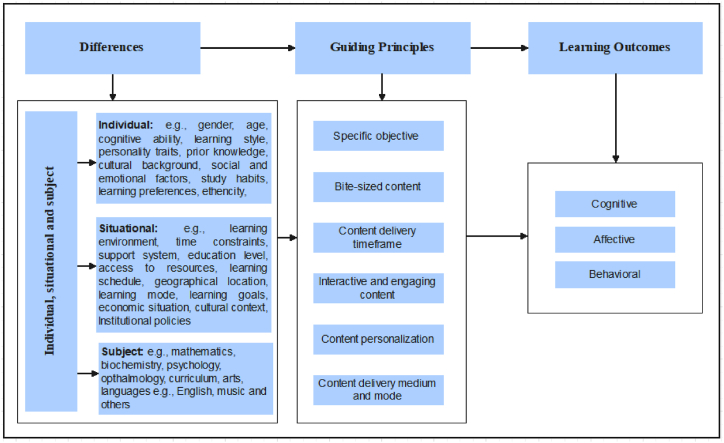
Proposed conceptual framework for microlearning instruction design.

The analysis revealed that microlearning has positive effects on learning outcomes (see Appendix B). In addition, several key themes emerged as beneficial for optimizing learning outcomes, labelled as guiding principles in this study. Based on the insights gained, we proposed a novel framework with three main components: (1) differences consisting of individual, situational, and subject differences; (2) guiding principles; and (3) learning outcomes (see Fig. 3). Each part of the Framework is described in detailed in the subsections.

#### 4.2.1 Part 1. individual, situational, and subject differences

Part 1 of the framework draws upon the Andragogy in Practice Model, particularly on the dimension of individual, situational, and subject differences [33]. Individual differences include characteristics such as gender, age, cognitive ability, learning style, personality traits, prior knowledge, cultural background, social and emotional factors, study habits, learning preferences, ethnicity, and others. Situational differences refer to contextual elements like the learning environment, time constraints, support systems, access to resources, learning schedule, geographical location, learning mode, economic situation, and institutional policies. Subject differences pertain to the specific areas of study, including fields such as mathematics, psychology, biochemistry, ophthalmology, curriculum design, arts, languages (e.g., English), music, and others. These factors influence the content and approach needed for effective learning in any discipline.

#### 4.2.2 Guiding principles

The key themes and sub-themes that emerged effective in improving learning outcomes are presented as guiding principles (see Table 4 and Fig. 3). Each one is described in detail below.

**Table 4:** Synthesis of the guiding principles of microlearning.

Studies	Themes	Description
e.g. Refs. [19,21,22,36,40,41,42,43]	Specific objective	Refers to addressing one particular concept, skill, or problem, enabling learners to grasp key information quickly and efficiently.
e.g. Refs. [17,18,19,20,22,23,36,40,44,46,47,48,51]	Bite-sized content	Refers to easily digestible units of content, promoting efficient learning and retention. The content is independent and self-contained, or part of a learning program allowing learners to build knowledge gradually.
e.g. Refs. [7,17,18,19,36,38,41,43,44,45,48,53,55]	Content delivery timeframe	Refers to determining the optimal duration of each learning module and the frequency of delivering these modules to balance effective learning, attention, and engagement without overwhelming the learners. Microlearning emphasizes shorter learning durations-seconds and/or minutes and just-in-time or need-based sessions.
e.g. Refs. [17,19,37,38,40,41,42,43,44,45,48,49,52,55]	Interactive and engaging content	Refers to experiences designed to be interactive and engaging, promoting deeper understanding. It incorporates interactive elements and multimedia to improve learner engagement, motivation, and retention of knowledge.
e.g. Refs. [37,38,41,42,52,53,56]	Content personalization	Microlearning provides individualized learning experiences by delivering targeted information aligned with specific learning objectives.
e.g. Refs.[19,22,23,[44], [45], [46], [47]]	Content delivery medium and mode	Refers to the selection of the appropriate medium and mode to deliver microlearning content to learners, considering factors like learning objectives, learner preferences, and the learning environment.

##### 4.2.2.1 Specific objective

Microlearning is characterized by its emphasis on a single, well-defined learning objective. The content is kept concise and to the point, allowing learners to quickly grasp the key concept. These objectives (typically one per module) are action-oriented, guiding the design and implementation of the modules, with an immediate focus on practical application. Each module objective is clearly defined and states indicating what learners should learn, or be able to do after engaging with the microlearning content. Several of the analyzed studies highlighted the significance of a single definable objective (e.g. a specific concept, problem, skill, topic, or other area) for microlearning outcomes [19,21,22,36,[40], [41], [42], [43]]. By keeping the objectives concise and well-defined, learners can better absorb and apply the concepts without feeling overwhelmed by excessive information, thereby reducing cognitive overload. McNeill and Fitch [42] highlight the possible issue of cognitive overload in microlearning when there are too many objectives, an excessive amount of content, or lengthy activities. A single objective in microlearning promotes efficient learning and improves retention. The focus on a single idea or topic distinguishes microlearning from other instructional methodologies [42] and allows for precise alignment with learners’ needs. Furthermore, well-defined objectives can be achievable within a short timeframe. Effective microlearning objectives are learner-centred and relevant, providing practical and applicable knowledge that can readily be applied in real-world contexts as Choo and Rahim [19] state that topics should be related to future factual work situations or objectives. A focused selection of topics and examples in microlearning minimizes arbitrariness by ensuring uniform, structured information delivery to all [7].

##### 4.2.2.2 Bite-sized content

The findings from the analyzed studies converge on the agreement that microlearning content should be short and focused, using various terms to describe it such as bite-sized [19,22,23], small chunk [42], segment [44], small burst [36], small units [38], and others. These terms collectively refer to the concept of breaking down information into more manageable pieces. Although there may be subtle differences in emphasis among these terms, the ultimate goal remains to create concise and easily comprehensible content to prevent overwhelming learners with cognitive load. The terms ‘short content’ or ‘small units’ used in the literature are more generic and may not capture the sense of quick, efficient, and focused learning that microlearning aims to achieve. Therefore, we choose to use ‘bite-sized content,’ or ‘small chunks’ instead of ‘short content’ or its alternatives which emphasizes the idea of breaking information into easily digestible pieces that can be consumed quickly. The term ‘bite-sized’ implies that the content is small enough to be consumed in a short period of time, just like taking a small bite of food. It conveys the notion of easy consumption and encourages learners to engage with the content without feeling overwhelmed. In microlearning, content developers assume a vital role, responsible for creating and reviewing effective learning materials. Their instructional design skills are important in crafting compact and user-friendly content [21]. An essential aspect highlighted is the need to chunk microlearning content to enable its stand-alone use while ensuring its seamless integration into a larger training series [21].

##### 4.2.2.3 Content delivery timeframe

Microlearning modules are designed to be short to prevent cognitive overload, enabling learners to complete them in a brief timeframe. The duration should provide sufficient information while allowing learners to process and apply the content effectively. However, the ideal length of microlearning content is subjective and not agreed upon as the included studies have various durations reported such as 1–3 min [44], 5–7 min [23], 5–8 min [45], 5–10 [22], and 10–15 min [46]. Nevertheless, studies suggest that the short duration of microlearning is more engaging and productive. For instance, a study conducted by Choo and Rahim [19] on pursuing new knowledge through microlearning found that 84 % of students were engaging with the short videos and spending a substantial amount of time watching them (over 80 % average views duration). The frequency of accessing classes, materials, or resources in microlearning is based on individual needs and preferences. That is microlearning is inherently need-driven and follows a just-in-time delivery strategy, allowing learners to acquire information or skills immediately when they need them. Learning tasks should be balanced to maintain interest. Microlearning, with content delivered in smaller units over an extended period, may be a more effective approach [47]. The study by Roskowski et al. [48] found improvement in learning, but it was stated that it is unclear if a longer period before assessing behaviour change or repetition of content would have resulted in a different outcomes.

##### 4.2.2.4 Interactive and engaging content

Microlearning effectiveness extends beyond content delivery; it hinges on student utilization and engagement [17]. In other words, it is not enough to have the content available – students need to actively use and engage with it for microlearning to be effective. To achieve optimal engagement, microlearning content should be visually appealing and interactive. Several studies reported its engaging nature (e.g., [[17], [18], [22], [23], [37], [40], [42], [44], [45], [48], [49]]). For instance, microlearning was found to stimulate critical thinking, and collaboration, promoting student-student interaction and comprehension of new concepts [37]. Strategies encompass using interactive elements, gamification, and social learning features [21]. The study conducted by Fidan [37] also found that microlearning content creates an engaging environment. Additionally, Ho et al. [17] found success in encouraging adult learners to engage with bite-sized learning units during designated time periods.

##### 4.2.2.5 Content personalization

Microlearning personalization refers to the process of tailoring microlearning content to meet the specific needs and preferences of learners. Personalization in microlearning involves adapting the content, format, and delivery of the learning materials to align with the individual learners' needs. This can include personalizing the content to reflect the learners’ interests, job roles, or skill levels. It may also involve adjusting the delivery method or format to accommodate different learning styles, or preferences, such as providing text-based materials, videos, quizzes, or interactive exercises. Customizing microlearning can improve learner engagement, improve knowledge retention, and increase the effectiveness of the learning experience. It allows learners to receive targeted and relevant information in a format that suits their learning needs, leading to more efficient and personalized learning outcomes. With its personalized and inherent flexible nature, microlearning has been well-received by participants with high satisfaction. For instance, Manning et al. [40] found that microlearning content was engaging and highly relevant for the participants. However, when the content becomes less relevant or feasible, learner engagement tends to decrease. For example, in Beste [7] study, participation in later modules decreased due to time constraints or relevance concerns. However, the series received positive feedback, with nearly 50 % completing all modules 75 % completing the first lesson and 91 % evaluating the course as relevant.

##### 4.2.2.6 Content delivery medium and mode

The analyzed studies utilized various media formats, platforms, and devices for delivering microlearning instruction content. They used a diverse range of media formats, including printed and non-printed [38]. The media elements, such as videos, podcasts, and flashcards, facilitate quick comprehension and retention of knowledge [37]. For example, Rahbar et al. [50] delivered microlearning materials through different formats, including ten 4-min audio podcasts on key diabetes management topics, twenty-five short text messages with reminders and tips, and twelve text messages with photos for improved engagement. In addition, incorporating interactive multimedia elements improves learner engagement, while students have shown a clear preference for video-based microlearning content [38]. Similarly, Shabadurai et al. [23] state that video is the primary and highly effective element for delivering microlearning content. Slideshows [45] and white drawings can also be used.

The studies used various platforms and apps including specialized, traditional, and social. They included Gnowbe [17], NeNA [21], Axonify [51], and EdApp [41] which are designed to host and deliver microlearning content to learners. Additionally, Learning Management Systems (LMSs) [36,37] like Moodle learning platform [45] were found to be used for microlearning. They often include features such as content creation tools, progress tracking, and analytics to monitor learners’ performance and engagement. The selection and utilization of appropriate technology platforms are vital for the successful implementation of microlearning interventions. In addition, social learning platforms such as Twitter (X) [47,52], Facebook [47], LinkedIn [52], TikTok [49] and Instagram can be used for microlearning delivery. The rapid adoption of social media platforms like Twitter and Facebook has opened up new pathways for communication using condensed content [47]. Learners favour interactive, visual-based microlearning content within a social learning setting, and the periodic sharing of bite-sized material via platforms like Edmodo served as a motivating factor [37].

Microlearning content was accessed through various devices. These include mobile devices such as smartphones and tablets [21,38,39,49,53], and computers [38]. These devices allow learners to access microlearning content on the go or in other different learning environments, promoting flexibility and convenience. Learners primarily learn microlearning content through personal mobile devices, promoting a seamless and convenient learning experience [21].

Regarding the modes of microlearning delivery, they vary including face-to-face, online, or blended approach [54]. Deciding on the microlearning mode is critical to align with the learning objectives, audience preferences, content complexity, resource availability, and overall learning experience. It is noteworthy, however, that these modes differ from traditional modes by being underpinned by the foundational principles of microlearning.

#### 4.2.3 Microlearning in improving learning outcomes

The effectiveness of microlearning in improving learning outcomes was consistently reported in the analyzed studies. The included studies demonstrate positive impact of microlearning on learning outcomes. These outcomes have been categorized according to Bloom's Taxonomy into three domains: cognitive (intellectual), affective (emotional), and psychomotor (behavioural) [57].

##### 4.2.3.1 Cognitive outcomes

The cognitive domain involves acquiring knowledge and intellectual skills [58]. Based on this domain, the findings indicate that microlearning leads to knowledge acquisition, improvement, transfer, and/or application (e.g., [[7], [19], [21], [22], [23], [36], [39], [40], [41], [42], [43], [44], [48], [51], [52], [59], [60], [61]]). In addition, microlearning resulted in improved information processing, retention, and/or recall (e.g., [[17], [19], [21], [23], [38], [40], [42], [44], [48], [59], [73]]), improved ability to apply new knowledge in related settings (e.g., [[19], [20], [40], [41], [42], [51], [60], [61]]). For instance, Manning et al. [40] highlighted the noteworthy impact of using a bite-sized teaching approach (BST), resulting in improved knowledge acquisition and immediate knowledge recall, outperforming traditional case-based teaching methods. Furthermore, microlearning was found effective in the development of self-regulation of learning and/or metacognitive skills [18,37] such as improved critical thinking, and/or problem-solving skills [37]. The improved skills also included peer feedback skills and second language skills or basic English communicative skills [53]; digital skills required for text processing, creating presentations, information seeking, and email management [38]; professional skills [41]; skills in developing learning goals and objectives [20]. The analysis also reported improved core competencies including digital competence and/or digital pedagogy [[62], [74]]; self-assessed ability to effectively create and use faculty development (FD) snippets [43], and competence in specific subject areas [49]. Performance included learning performance and test performance such as improved scores [[19], [72]], improvement from unit to unit [53], and final exam scores or overall academic achievement [63]. For example, Roskowski et al. [48] observed that both microlearning and the traditional method improved knowledge and self-efficacy scores compared to the baseline. However, the majority of participants expressed a strong preference for microlearning over the traditional method, underscoring its appeal and effectiveness. In another study by Choo and Rahim [19], knowledge was found to be comparable in online distance micro-learning and traditional face-to-face active learning, suggesting that microlearning can be just as effective as traditional methods. Some of these studies focused on technology-oriented microlearning and demonstrated improved outcomes in related skills and competencies [e.g., 41, 49, 53]. For instance, a TikTok-based microlearning environment received positive feedback on content and competencies [49], the Pebasco mobile microlearning app improved peer feedback skills and L2 proficiency [53], and the mobile microcourse improved learners' confidence and achieved its learning effectiveness goals [41]. Microlearning proved effective in virtual environments. For instance, Choo and Rahim [19] studied pharmacy students’ perceptions and performance in a microlearning-based virtual practical on the elucidation of drug absolute configuration. The study found that the virtual micro-learning design was practical and economical, requiring minimal technology and training, and was well-suited for open distance learning environments, especially post-COVID. Furthermore, microlearning integrated into other teaching methods, such as microlearning flipped classroom (m-FC), improved learning performance compared to non-flipped classroom settings [37].

##### 4.2.3.2 Behavioural outcomes

The psychomotor (behaviour) domain involves acquiring manual and physical skills [58] and actual behaviours occur [64]. The outcomes included presentation skills and teaching skills [40]; and practical performance such as work performance [53]. In the study by Manning et al. [40], the majority of residents who participated as bite-sized teaching (BST) speakers reported that the experience helped develop presentation skills and intended to apply the learned skills in their own teaching. In another study on the NeNA microlearning app, improved job performance, socialized learning, and personal growth were revealed [21]. Microlearning also led to higher completion rates [59], and engagement such as involvement in-class activities, an active role in the learning process [18], contribution, collaboration [17,18,21,22,37,40,45,48,49], and/or usage [12,17,18,37,43,56,60]. For instance, Hegerius et al. [61] found that almost all learners (98.5 %) expressed the intention to put into practice what they had learned through microlearning. Furthermore, it included behavioural patterns (e.g., correct answer requests, top audio) [53]; learning days (time between first and last login), number of events (clicks, text written, and other student input), the total number of visits, length of each visit, type of device (smartphone, tablet, desktop computer), and course components used by students [38]; helping behaviour [59]; perceptive behaviour [48].

##### 4.2.3.3 Affective outcomes

The affective domain involves integrating beliefs and ideas (Attitudes) [58]. This domain focuses on attitudes, emotions, feelings, and values, rather than on knowledge and skills. Learning outcomes found related to this category, included increased motivation in learning activities [7,17,[21], [22], [23],^37^,^40^,^45^,^49^,^52^], such as intrinsic motivation (e.g., learning as a hobby) and extrinsic motivation (e.g., learning digital marketing to get a promotion) [65], enjoyment [19], interest and goal achievement [47], and encouragement [18]. Furthermore, the analyzed studies reported increased satisfaction [17,18,20,36,38,48,49,51,[59], [60], [61], [75]]. For instance, Hegerius et al. [61] evaluated microlearning-based modules developed and found that participants were highly satisfied with the modules, with nearly all recommending them to colleagues, noting their effectiveness in improving knowledge and skills. Microlearning also improved self-efficacy, confidence, and/or social connectedness) in the subject matter [18,19,37,41,45,48,60], positive attitudes or perceptions and feelings toward the learning content [7,[38], [39], [40],^47^,^56^,^59^,^60^]. Examples included favourable comments and well-received feedback [19], and positive reactions [51]. The approach contributed to positive learning experiences [37,44,63]. Participants had a sense of belonging and social connectedness through specifically designed platforms such as NeNa, and Edmodo or social media such as Facebook, LinkedIn, and Twitter [18,21,23,37,[39], [40], [41],^49^,^51^,^52^,^59^], development of ethical and moral values or reasoning in decision making [39], and heightened willingness or curiosity about content or participation [37,49]. Microlearning was found to be effective [18,38], with the potential to contribute significantly [56] and offer both effectiveness and efficiency [48], reduced anxiety and stress [18,46] and perceived relevant.

## 5 Discussion

### 5.1 RQ1. How is the definition of microlearning and its didactic conceptualized in the selected studies?

The findings of this SLR substantiate and strengthen the existing body of research, affirming the core understanding of microlearning as an instructional approach that effectively delivers bite-sized targeted learning content to accomplish specific objectives. ‘Approach’ was the most dominant didactic used in the selected studies. This finding is consistent with the review of Nikkhoo et al. [66], who emphasize that microlearning is an engaging educational approach that promotes the acquisition of new skills and information through the use of small learning parts at a time. Similarly, Prior Filipe et al. [20] state that it is an effective instructional approach that delivers concise and targeted learning units through short-term activities, focusing on specific skills or knowledge areas. Microlearning is considered need-based, specific-objective driven, action-oriented, self-contained, and flexible (accessible anytime, anywhere, and through any learning tool).

However, depending on the specific aspects being emphasized, ‘microlearning’ was also referred to using various didactics such as instructional method (e.g., [[18], [20], [40]]), technique [22], learning tool [22], pedagogy [47], strategy (e.g., [[40], [43], [48]]), learning format [20,43], concept [67], module [46] or model [43]. Other reviews such as that of Leong et al. [5] describe microlearning as a new topic, while Sankaranarayanan et al. [11] consider it as a format of learning. This diverse range of terminology and didactic demonstrates the various dimensions and perspectives associated with the microlearning approach. Each term emphasizes a distinct aspect of microlearning enriching our understanding of how microlearning is conceptualized and implemented in different educational contexts. The authors have differing views regarding microlearning, and its spelling varies (e.g., ‘microlearning,’ ‘micro-learning,’ ‘micro learning,’ ‘MicroLearning’). ‘Microlearning’ is commonly used, showing broad acceptance, while ‘micro-learning’ and the other alternatives might be used per style guides. Consistence is important in its usage as it improves uniformity, clarity, and understanding. With regard to the wide usage of ‘microlearning,’ similar findings were also observed in a scoping review conducted by Taylor and Hung [8]. Other nomenclatures (‘bite-sized learning,’ ‘snippet learning,’ ‘just-in-time learning or teaching’) help capture the various ways it can be applied and understood in diverse contexts. The unified use of ‘microlearning’ underscores its established role, while varied terms may reflect its evolving nature. This reflects its novelty in education and corporate training [7,51].

Microlearning seems to have emerged primarily as a response to the evolving demands of modern education and training, particularly within the context of the digital age. The rise of technology and the increasing prevalence of eLearning, mobile learning, and informal learning environments have played a crucial role in its development and adoption. As learners increasingly prefer personalized, on-demand learning, microlearning is likely to remain sustainable and continue growing.

### 5.2 RQ2. What are the effects of microlearning on learning outcomes?

The findings from the analyzed studies revealed that microlearning is effective in improving learning outcomes. Key factors that may have contributed to the improved learning outcomes were identified and synthesized. The analysis led to the development of a novel instructional design framework comprising three main components: (1) differences consisting of individual, situational, and subject differences; (2) guiding principles; and (3) learning outcomes.

#### 5.2.1 Individual, situational, and subject differences

This part of the framework draws upon the Andragogy in Practice Model [33]. Individual factors such as gender, age, and cognitive ability can shape the design of microlearning content. For instance, Javorcik et al. [38] study found that men studied significantly longer, surpassing women in study duration. In another study by Iqbal et al. [60] who investigated perceptions of residents regarding the microlearning environment in postgraduate clinical training found that while residents generally had positive perceptions and satisfaction with the microlearning environment, female residents and those aged 25–30 reported higher satisfaction. Modules can be adapted to offer content of varying complexity, ensuring relevance for a broad age range. For example, a language learning app might provide basic vocabulary exercises for younger users and more advanced grammar challenges for adults. This adaptability acknowledges diverse cognitive levels. Similarly, adaptability extends to personality traits, with extroverts favouring group activities and introverts leaning toward solitary reflection. Learning styles, such as visual learners benefit from videos, interactive quizzes engage kinaesthetic learners, and concise audio suits auditory learners. The study by Javorcik et al. [38] revealed that students preferred videos and video tutorials over text materials, with preferences varying according to the chapter thematic focus. Another critical consideration is prior knowledge which influences the learning process, serving as a foundation upon which new information is built. For instance, in language learning, individuals with prior exposure to related languages might grasp new vocabulary and grammar rules quickly. Microlearning needs to integrate culturally relevant examples for diverse learner engagement. In addition, social and emotional aspects are pivotal, with instant feedback and gamification addressing emotional needs and improve motivation through rewards. For example, gamified elements, such as earning points or badges, inspire motivation and create a sense of achievement.

To effectively address situational differences, microlearning must be adaptable, accessible, and responsive to the dynamic learning environment, time constraints, support systems, access to resources, and more. For example, McKee and Ntokos [45] examined online microlearning and student engagement in computer games in higher education. They found significant variation by year of study: first-year students preferred videos lasting 6–12 min, whereas second and third-year students preferred videos under 8 min. Overall, engagement was highest with videos lasting 5–8 min. Situational differences may influence the effectiveness of microlearning, shaping its design to cater to learners' unique circumstances. The learning environment plays a pivotal role; whether it is a classroom, online platform, or workplace setting, microlearning can be adapted to the context. Microlearning concise format suits busy times, allowing learners to fit short modules into their daily routines. Flexibility in scheduling is essential, accommodating those with limited availability. Learners' access to instructors, peers, or online communities provides opportunities for discussions, clarifications, and knowledge sharing. Incorporating such support mechanisms ensures that learners never feel isolated, fostering a sense of engagement and belonging. Making microlearning accessible regardless of learners’ physical location is also significant. Microlearning aids in facilitating adaptable digital learning across geographically dispersed locations [7]. In addition, institutional policies play a role in facilitating or hindering microlearning adoption. Alignment with institutional guidelines and regulations ensures smooth integration and optimal utilization of microlearning.

In terms of subject differences, it can significantly impact the design, implementation, and effectiveness of microlearning. While microlearning can simplify complex topics, the approach will differ based on the subject intricacy. For example, Conde-Caballero et al. [49] found significant differences in the effectiveness of microlearning based on the subject in which it was deployed. The development of the module should consider whether the content is theoretical, practical, or complex, even content within the same subject can vary in complexity. For example. in a complex topic such as English language verb tenses due to the various forms and usage rules, the microlearning module could provide concise explanations of verb tense (e.g., present, past, future), and offer real-life examples in sentences. Visual aids, such as timeline diagrams or charts, could help learners visualize the progression of time and tense changes. For a simpler English language topic like ‘Basic Vocabulary: Colours,’ the microlearning content might include short audio clips pronouncing different colours, accompanied by colourful visuals of each colour name. Interactive drag-and-drop activities could reinforce learners' ability to match the word with relevant colours. In these instances, microlearning aligns with the subject complexity, ensuring learners understand the fundamental concepts and apply them in practical language use.

#### 5.2.2 Guiding principles

The common practices were identified from the analyzed studies that may have contributed to effective microlearning. These practices were then synthesized into specific themes labelled as guiding principles. One of the crucial principles is choosing a specific objective that is action-oriented, guiding the design and implementation of microlearning module, with an immediate focus on practical application. A single definable objective can be a specific concept, problem, skill, or other area [21,22,36,[40], [41], [42], [43]].

By keeping the objectives concise and well-defined, learners can better absorb and apply the key concepts without feeling overwhelmed by excessive information, thereby reducing cognitive overload. McNeill and Fitch [42] highlight the possible issue of cognitive overload in microlearning when there are too many objectives, an excessive amount of content, or lengthy microlearning activities. The objective should be learner-centred, relevant, and practical in real-world contexts. For example, a single objective, such as performing CPR in an emergency, may be targeted in the microlearning module. The relevant content guides the steps of CPR through interactive simulations, allowing learners to practice the correct rhythm and compression depth on a virtual mannequin, thereby reinforcing life-saving skills. The content should be concise, often described as ‘bite-sized,’ ‘small chunk,’ or ‘nugget’ to prevent cognitive overload. Chunking content is crucial for both standalone use and integration into broader training. For example, extensive learning materials can be condensed into bite-sized materials which can be managed to be delivered through short media such as videos (1–3 min) and can even be conveyed through single-sheet infographics [44]. As the world is rapidly evolving, embracing advanced technology in microlearning is vital. Cutting-edge technologies, such as generative AI, can particularly help in condensing large amounts of learning materials into bite-sized formats. For instance, lengthy documents can be summarized by Generative AI tools, making the content easier to understand, retain, and utilize. These can be delivered through varied delivery modes including face-to-face, online, and blended [54].

The timeframe of bite-sized content is subjective and there is an ongoing debate about the optimal duration of microlearning lessons, with reported ranges varying from a few seconds to a few minutes [e.g., 23, 44, 46]. However, the short duration is prioritized to prevent cognitive overload. Microlearning, with content delivered in smaller units over an extended period, may be a more effective approach [47]. For instance, in the Choo and Rahim [19] study, the recorded demonstrations uploaded as videos on YouTube ranged from 12 to 62 s. Extended explanations were offered through longer videos: an introductory video (9:27 min) and a ‘Question 1’ video (2:34 min) explaining the R and S assignment. These videos, along with quizzes, were embedded into a newly designed Google Form (GF). Students had a week to complete the assessment, featuring 11 sections with 55 questions translated from a face-to-face practical session.

Microlearning effectiveness extends beyond content timeframe and delivery; it hinges on student utilization and engagement [17]. To achieve optimal engagement, microlearning content should be visually appealing and interactive. Strategies encompass interactive elements, gamification, social learning features [21], simulation, interactive flashcards, and feedback mechanisms such as instant feedback and peer feedback. The study conducted by Fidan [37] found that microlearning creates an engaging environment, offering numerous opportunities to foster critical thinking, student-student interaction, collaborative learning, and the understanding of new concepts or subjects, while Ho et al. [17] found success in encouraging adult learners to engage with bite-sized learning units during the designated time.

Personalization in microlearning involves adapting the content, format, and delivery of the learning materials to align with the individual learner's requirements. This can include personalizing the content to reflect the learner's interests, job role, or skill level. It may also involve adjusting the delivery method or format to accommodate different learning styles [68], or preferences, such as providing text-based materials, videos, quizzes, or interactive exercises. It allows learners to receive targeted and relevant information in a format that meets their learning needs, ultimately leading to more efficient and personalized learning outcomes.

In terms of medium, the findings indicate that microlearning can be delivered through a variety of tools including various media formats, platforms, and devices. The media format can be videos, podcasts, and text, fostering rapid understanding and retention [23,37] with videos as an effective medium for delivering microlearning content [23]. For instance, the Choo and Rahim [19] study delivered micro-content through short videos that highlight specific aspects of an earlier lecture, where the lecturer demonstrated 3D molecular structures of a chemical substance using molecular model kits. These demonstrations were recorded, trimmed, and uploaded as videos on YouTube. Innovative technologies like generative AI can be used to create microlearning content in various formats such as animated videos, graphs, and text. The platforms include specialized ones like EdApp [41], Learning Management Systems (LMSs) [36,37] like Moodle [45], and social media platforms like TikTok [49]. Bannister et al. [52] exemplified this by converting educational case study clinic and podcast segments into concise 30-180-s media clips, tailor-made for standalone microlearning content on social media. The distribution strategy involved posting these microlearning units on both Twitter and LinkedIn, with a consistent frequency of two posts per platform per week, spanning three months. As most platforms have integrated Generative AI, they can be employed to further optimize content delivery by offering real-time assistance, language translations, or adaptive pacing based on learner behaviour. The available content can be accessed through various devices including mobile devices like smartphones and tablets [21,38,49] as well as computers [38,55,69]. These devices enable learners to access microlearning content at their convenience, with personal mobile devices being the primary means of engagement [21]. For example, Lee et al. [41] designed a mobile microcourse, using a comprehensive three-stage development process for effective microlearning delivery. Initial microlessons were crafted by an expert journalist using EdApp, and subsequently refined through expert and researcher reviews. User input from a pilot test further honed the course, leading to the creation of ‘The 5 Cs of Writing News for Mobile Audiences.’ This course specifically caters to journalism students and professionals seeking to elevate their news writing capabilities for mobile readers.

The prepared microlearning content can be delivered through various modes. Selecting the appropriate mode is critical and can be influenced by factors such as learner preferences, content complexity, and available resources. For instance, some learners may prefer face-to-face interactions, while others may prioritize the flexibility of online microlearning. With respect to complex content that necessitates high interactivity face-to-face delivery may be better suited, facilitating discussions, and personalized guidance. Alternatively, they may be more suitable for online asynchronous microlearning, such as short iterative videos. Blended microlearning, combining face-to-face and online elements, offers a harmonious way. It fosters interactive in-person learning and sustains reinforcement through online modules. For instance, Ho et al. [17] in the 6-week Cognitive Psychology course, a blended course, 3 weeks of face-to-face sessions, and 3 weeks of online learning investigated microlearning. Bite-sized learning activities were introduced 10 days in advance to act as catalysts for upcoming topics for face-to-face sessions. These activities, designed as pre-class tasks, not only engaged learners but also facilitated concept exploration. Subsequently, the instructor expanded on these concepts during in-person sessions, explicitly establishing the connections between bite-sized activities and classroom teachings. Another important factor is resource availability, encompassing time, budget, and technology which can influence the mode chosen.

#### 5.2.3 Learning outcomes

To assess learning outcomes aligned with Bloom's Taxonomy, evaluation should be tailored to the specific domain of learning (cognitive, behavioral, or affective) being addressed. Each domain represents a different aspect of learning, encompassing knowledge, skills, and attitudes. The cognitive domain focuses on intellectual abilities, knowledge acquisition, and thinking processes. Cognitive learning outcomes involve various levels of understanding and application of concepts related to the subject matter. Examples include knowledge acquisition, improvement, transfer, and application; improved critical thinking and problem-solving skills; improved information processing, retention, and recall; development of metacognitive skills, and self-regulation of learning. Sankaranarayanan et al. [11] highlight that microlearning allows for the efficient acquisition and application of knowledge and skills. Similarly, in a systematic review and meta-analysis, Prasittichok and Smithsarakarn [25] found that microlearning significantly outperformed traditional lectures in improving English-speaking skills for EFL students. The findings emphasize microlearning effectiveness in English language teaching and improving EFL speaking skills. In another study, Wang et al. [26] found in their SLR that microlearning can improve cognitive self-care abilities and, under conditions, can also be effective in improving actual self-care behaviours.

The behavioural domain focuses on the development of manual and physical skills [58] and actual behaviours [64]. Learning outcomes in this domain pertain to observable behaviours and actions that learners can demonstrate. Behavioural outcomes include the development of psychomotor skills, task completion, improved performance, engagement, contribution, collaboration, and utilization. De Gagne et al. [70] in their review reported that microlearning positively affected health professions students’ engagement in collaborative learning, alongside their knowledge, confidence in performing procedures, knowledge retention, and studying.

The affective domain focuses on attitudes, emotions, and values. Learning outcomes in this domain involve changes in learners’ feelings, motivations, and beliefs. In microlearning, affective outcomes included increased motivation—both intrinsic (learning as a hobby) and extrinsic (learning for career advancement). Satisfaction, self-efficacy, and positive attitudes toward microlearning were frequently reported. Other examples include increased confidence, a sense of belonging and social connectedness, development of ethical and moral values or reasoning in decision-making.

## 6 Implications

The findings from this study have both theoretical and practical implications. The study reinforces the preference for ‘microlearning’ as the established term, ‘approach’ as the favoured didactics, ‘bite-sized content’ as the preferred measure for brief instructional segments, and ‘seconds or a few minutes’ as the delivery time. Therefore, microlearning can be defined as an instructional approach focused on delivering bite-sized, targeted content to diverse learners within seconds or a few minutes. This approach aligns closely with Sweller's CLT. The theory suggests that breaking information into manageable chunks reduces cognitive overload and improves learning efficiency. The alignment between microlearning and CLT underscores its effectiveness in optimizing cognitive processing and retention. The use of microlearning as a practical application of CLT could lead to further exploration and validation of the theory's principles in different learning contexts. Additionally, the study contributes to the understanding of Andragogy by highlighting individual, situational, and subjective differences observed in the analyzed studies.

In terms of practical implications, a microlearning instruction design framework is proposed for its future implementation, encompassing three main dimensions including differences, guiding principles, and learning outcomes. Educators, instruction designers, and microlearning educational app developers can utilize this framework to design and deliver effective micro modules. For instance, by focusing on factors such as individual, situational, and subject differences and the guiding principles during micro module design and delivery, educators can create more effective and personalized learning modules. They should consider the guiding principles. First, microlearning module is specific-objective driven, ensuring a clear focus on specific learning outcomes. Second, the content is delivered in bite-sized portions, allowing learners to grasp information efficiently and effectively. Third, careful consideration of the duration and frequency of microlearning activities, can optimize knowledge retention and application. Fourth, interactive and engaging activities should be incorporated to foster active engagement. Fifth, personalization is to cater to individual learning needs and preferences. Lastly, the selection of appropriate instruction delivery medium and mode diversifies the learning experience, accommodating various learning preferences.

The potential for microlearning to improve cognitive, behavioural, and affective learning outcomes suggests that it can be a valuable approach to learning. It can be used for improving learners’ specific cognitive outcomes (e.g., knowledge), behavioural outcomes (e.g., engagement), and affective outcomes (e.g., motivation). For instance, instead of a full-day workshop on company policies, employees receive brief, 5-10-min micro modules in the form of video tutorials on specific topics like compliance, IT security, or company values. This reduces cognitive overload, allowing learners to better retain important information and gradually build their knowledge base.

## 7 Limitations and future recommendations

While this study provides valuable insights, it has several limitations. First, the study was limited to microlearning outcomes. Future research should address meso and macro-level learning outcomes. Research should also extend to exploring microlearning across diverse educational and cultural contexts to capture a broader spectrum of applications and outcomes. Second, the date range of 2020–2024 may have excluded relevant research outside this timeframe. Future reviews could use AI-powered tools to include and analyze relevant literature more comprehensively, providing a richer perspective. Additionally, the study only included articles published in English, thereby excluding those in other languages. The study focused solely on peer-reviewed academic journal articles, excluding other forms of grey literature where relevant reports on microlearning might also exist. Although a sufficient range of search terms was used, the defined and keyworded search terms might still affect comprehensiveness. Using specific keywords in search engines may risk excluding relevant literature. Future research could explore alternative synonyms to capture a broader range of papers describing microlearning. Decisions about language and meaning in the framework may introduce bias, as researchers interpret the language used by the original authors. We aimed to limit subjectivity by involving two coders and double-checking the process with co-authors. The framework can be used as a basis for developing more comprehensive theoretical models that integrate microlearning with other instructional strategies and educational theories. The framework needs to be tested for validity and reliability.

## 8 Conclusion

This study adds to the ongoing discussion on the definition and didactics of microlearning, and its effects on learning outcomes. Findings from the analyzed studies reveal that microlearning is an effective instructional approach that delivers bite-sized, targeted instructional content within a short period, typically in seconds or a few minutes. This specific objective-driven approach aligns with CLT and learner-centred principles. Regarding the characteristics of the studies, most of them were conducted in 2022 and focused on medical fields, with a significant portion originating from the USA. The predominant use of quantitative methods was evident, with most research targeting learners and utilizing a combination of data collection tools. The key findings indicate that microlearning can potentially improve learning outcomes in all three domains including cognitive, behavioural, and affective. In addition, the proposed instructional design framework consisting of three integral components—differences including individual, situational, and subject differences; the key guiding principles; and learning outcomes—provides educators and curriculum designers with valuable guidance to effectively design and implement microlearning for improving learning outcomes.

## CRediT authorship contribution statement

**Wali Khan Monib:** Writing – original draft, Visualization, Methodology, Formal analysis, Data curation, Conceptualization. **Atika Qazi:** Writing – review & editing, Validation, Supervision, Software, Resources, Methodology, Funding acquisition, Conceptualization. **Rosyzie Anna Apong:** Writing – review & editing, Validation, Supervision, Resources, Methodology, Conceptualization.

## Data availability statement

Not Applicable.

## Funding statement

The work is supported by 10.13039/100009100Universiti Brunei Darussalam under research grant 10.13039/100009100UBD/RSCH/URG/2024/012.

## Declaration of competing interest

The authors declare that they have no known competing financial interests or personal relationships that could have appeared to influence the work reported in this paper.

## Figures and Tables

**Table:** 

**Author(S) & Year**	**Country**	**Field of study**	**Research Question(s)/objectives**	**Method**	**Population**	**Sample size**	**Research Instrument**	**Findings**
[71]	USA	Computer science and information technology, online introductory database programming class	To explore the influence of the microlearning instructional approach in an online introductory database programming classroom	Exploratory case study	Students and instructor	Student (33), instructor (1)	Quizzes, exams, reflection prompts, perception survey, interviews	Learners scored significantly higher, experienced less cognitive load, and were more motivated and engaged. Microlearning was highly useful, contextually relevant, accessible, and flexible, leading to better knowledge retention. However, issues included fragmented learning experiences, the need for relevant feedback, and difficulties in learning without prior background knowledge.
[72]	India	MBA program	To evaluate microlearning effectiveness.	Experimental research, post-test control group design	Students	104	Quizzes and reaction survey	Students in the microlearning module scored significantly higher and showed improved performance and satisfaction compared to those in the regular module.
[50]	Iran	Diabetes Clinic	to investigate the impact of social media-based microlearning (SMBM) on enhancing the knowledge, self-care, and self-efficacy behaviours	Pre-test-post-test intervention	Patients	80	Questionnaires	The social media-based microlearning (SMBM) appears to be an effective tool for improving self-efficacy, self-care, and knowledge among patients with type 2 diabetes.
[62]	Hong Kong	English language teaching context	To explore teacher professional development (TPD) for online and blended learning (OBL) via microlearning	Exploratory	Teachers	Survey (67) and interview (12)	Survey and interview	Microlearning was perceived as flexible and stress-free and allowed teachers to focus on relevant and immediate tasks to achieve digital growth, with the skills necessary to teach effectively in the context of the ‘new normal’; development of teachers' technological competence and digital pedagogy
[75]	UAE	Instructional technology course	To assess the impact of an innovative microlearning module on postgraduate students' perceptions and academic progress	Quasi-experiment	Students	100	Pre and post-test/Achievement test, questionnaire	Students expressed general satisfaction with using the module during their learning and flipped microlearning courses. The ANCOVA test revealed a significant difference between the two groups, with an F value of 4.427 favouring the experimental group and a significance level of 0.038. Additionally, the questionnaire findings indicated a high level of student acceptance of the module.
[15]	–	Education Technology	Does the micro-reading activity promote students' reading effectiveness? Which micro-reading style can achieve better reading effectiveness?	Random study	Students	30	Formative and summative evaluation	The results showed that all students met the reading objective and maintained high engagement in the micro-reading activity. Summative evaluation revealed both groups had high average scores, but the electronic book reading (e-reading) group outperformed the paper book reading (p-reading) group, with significant differences noted. Thus, the 6-week micro-reading activity improved reading effectiveness, with e-reading proving more effective.
[65]	Spain	Business	To uncover student satisfaction and learning effectiveness of microlearning	Qualitative, case study	Students, experts, and company	Student (12), experts (4), company (2)	Primary source: individual interviews, quantitative and qualitative questionnaires, documentary reviews, and observations; secondary sources: public data (website, press news, Google, social networks)	Findings reveal that learning effectiveness is influenced by the training's relevance to students' jobs, leading to four outcomes: entertainment, knowledge updating, unexpected learning, and effective learning. This paper addresses gaps in research on learner satisfaction and microlearning effectiveness, emphasizing that these are not guaranteed.
[73]	Bulgaria	Orthopaedics and Traumatology	To compare the effectiveness of microlearning alongside traditional teaching methods	Random control study	Students	150	Mid and final-semester tests	Microlearning significantly improved students' knowledge retention, practical skills, and overall performance compared to traditional methods. Integrating microlearning into orthopaedics and traumatology curricula can improve learning outcomes and better prepare students for real-world practice.
[63]	Saudi Arabia	Education	Examines the effects of a Flipped Mobile-Based Microlearning (FMM) approach on postgraduate students' accessibility, engagement, retention, learning experience, and academic achievement.	Quantitative multiple methods approach	Students	19	Survey questionnaire	The results suggest that the Flipped Mobile-Based Microlearning (FMM) approach may positively affect accessibility, engagement, retention, learning experience, and exam scores compared to traditional methods. The findings support FMM's efficacy, highlighting its potential to improve learning and academic outcomes and underscore its value in educational practice and research.
[74]	Malaysia	Medical	Describes the implementation and preliminary evaluation of a competency-based virtual consultation training module for undergraduate medical students.	–	Students and alumni	52	Pre-test and post-test, quizzes, feedback, checklist	Microlearning and simulations effectively enhanced virtual consultation competencies in medical students.
[53]	Japan	CLT-focused EFL course/faculty of letters	To evaluate how an asynchronous microlearning app might improve students' skills for providing peer feedback on spoken content in the context of English as a foreign language (EFL) education.	Quantitative	Students	87	The Pebasco app	Pebasco (mobile microlearning app) indicates that many participants improved or maintained desirable patterns of behaviour and outcomes, suggesting a positive impact on the quality of peer feedback skills and second-language (L2) skills.
[38]	Czech Republic	Pedagogy	To determine if there are differences in approaches to studying a microlearning course	Quantitative	Students	First phase (378) + second phase (156)	Survey/questionnaire, learning analytics	Six different approaches lead to the successful completion of ML course. Gender is not influenced by the choice of strategies. The number of days to complete the course was the significant parameter. Detailed descriptions of learners' study strategies in ML courses can improve the effectiveness of ML. Students preferred videos and video tutorials over text materials, with the use of educational materials influenced by the chapter's thematic focus.
[56]	Netherlands	Hospital and homecare	Development of a microlearning intervention regarding nursing nutritional care for older adults	A multi-methods approach	Older adults, nurses, nursing assistants, experts, and other professionals involved in nutritional car	–	30 statements	Based on a well-founded and comprehensive procedure, the microlearning intervention was developed. This intervention has the potential to contribute to nursing nutritional care for older adults.
[48]	USA	Pharmacy	To evaluate microlearning as a preceptor development method compared to a traditional method of learning.	Quantitative and qualitative/crossover-controlled study	Preceptors	531	Pre and post-test, 4-point Likert scale questionnaire	Primary outcomes were satisfaction, changes in knowledge, self-efficacy, perception of behaviour, confidence scale, and self-reported frequency of behaviour, respectively. Most participants preferred microlearning over the traditional method. Free text satisfaction responses were analyzed using inductive coding and thematic analysis. Participants reported that microlearning was more engaging and efficient. There were no significant differences in knowledge, self-efficacy, or perception of behaviour between microlearning and the traditional method.
[37]	Turkey	Education	Explores the effects of the microlearning-supported FC on PTs' learning performance for professional development, motivation, and engagement.	Mixed method	Students	128	Quasi-experiment pre-test and post-test, questionnaires, and interview	The findings indicated that FC model (in both experimental groups) increased learning performance, intrinsic motivation, and emotional, and behavioural engagement compared to a group that was not used to FC. Moreover, m-FC group had better intrinsic motivation and engagement than both t-FC and non-FC groups. Most PTs had positive perceptions that it improves the willingness to participate in pre-class activities.
[49]	Spain	Nursing	Shared pedagogical insights from implementing a TikTok-based microcontent program across nursing degree modules	Quantitative and qualitative/longitudinal quasi-experimental	Students	213	Questionnaires	The findings indicate high satisfaction levels in terms of engagement, content quality, and technology acceptance. While gender-specific differences were not observed, slight variations were noted based on the subject where the microlearning tool was implemented.
[21]	USA	Military	design NeNA content	Quantitative	Employees/user	108	Two survey/questionnaire	The microlearning mobile app was effective in enhancing the performance and support of NATO employees. The app provided quick access to relevant information and training material, and the user feedback helped to improve the design of the app
[47]	USA	Workplace	Compare the effectiveness of three different interventions (face-to-face, online, and micro-learning) in developing psychological capital in individuals	Quantitative	Employees	228	Questionnaire	Supports all three modes but highlights notable advantages/effectiveness of online and microlearning.
[59]	Japan	Workplace	To evaluate the microlearning program effectiveness	Quantitative	Workers	150	Survey/questionnaire	The process evaluations confirmed high satisfaction and completion rates of the program among participants. The intervention group exhibited significant improvements in attitude and knowledge compared to the control group after 1 month. All outcomes remained significantly improved after 4 months.
[45]	UK	Bachelor of Arts and Bachelor of Science	Online microlearning and student engagement in computer games higher education	Quantitative	Students	94	Questionnaire	Engagement favoured an optimal range of 5–8 min overall. Significant variation emerged based on the year of study: first years preferred videos lasting 6–12 min, while second and third years leaned towards videos under 8 min.
[42]	USA	Education	To explore how learners experience online microlearning activities	Qualitative	Students	12	Quiz, Focus group interview	Eight Gagnies' event was found: gain attention, explain objectives, stimulate recall of prior learning, present the content, provide learning guidance, elicit performance, provide feedback, assess performance, improve retention and transfer, trust in content, design principle, purpose, and application.
[22]	Malaysia	Accounting	Investigating the impact of microlearning on student participation and learning interest	Qualitative	Students	40	Rubric assessments and observations, checklists and reflections	The outcome of the study showed that students' participation is at an excellent level. Micro learning has helped boost the interest of students in their learning.
[23]	Malaysia	Education industries	To identify the perspective towards microlearning and the significance of the design of micro-sized content for online training for employees	Quantitative and Qualitative	Employees: role of both trainers and trainees	190	Survey and focus group interview	Beyond videos, employees lacked awareness of other microlearning content elements for design. Two distinct architectures, namely Dual Architecture and Integration Domain Architecture, are introduced. Dual Architecture offers topics learnable through microlearning content with set durations or unrestricted gamified content. Integration Domain Architecture combines learning and evaluation, with each learned topic assessed through gamification techniques from the evaluation domain.
[12]	UK	Business	Aims to explore how learners prefer to interact with microlearning videos; in what ways do learners prefer to interact with multimedia microlearning?	Quantitative	Students	77	Questionnaire	The control function and expression functions of multimedia ML was high and perceived as useful. More respondents preferred to have more control on multiple-choice and open questions. No difference was found in the preference of the participants on when to attempt the assessment.
[44]	Indonesia	Curriculum development courses	To explore the effectiveness of microlearning as a mode of asynchronous learning in online learning.	Qualitative	Students	45	Questionnaire, document analysis, and interview	Microlearning effectively mediates cognitive load (intrinsic, extrinsic, and germane) in online curriculum development. ML usage is flexible and user-friendly in the online setting, leading to excellent learning outcomes. Most students hold positive perceptions and endorse microlearning's utility. They find the content easy to comprehend, adaptable to their learning style and appreciate its various formats. Asynchronous online activities support independent learning, granting easy access, control, and repetition of quality content. The presence of narration aids in material understanding.
[39]	Pakistan	Medical and Nursing	To test the effectiveness and feasibility of a mobile just-in-time learning (m-JiTL) method for bioethics education	Mixed methods approach	Students, residents, and practicing nurses	29	Pre-and post-questionnaire and knowledge tests, discussion logs, focus group discussions, document review, and open-ended questions	Bioethics knowledge test scores displayed improvement post-intervention, particularly evident among medical students. EthAKUL, the pioneering bioethics teaching app, provided valuable insights into novel pedagogies and technological integration for bioethics education. The study also highlighted design concerns in the app and m-JiTL pedagogy that require resolution before wider curriculum implementation.
[17]	Singapore	Cognitive psychology course	To examine whether adult learners utilized bite-sized learning activities during pockets of ‘down time’ in their busy schedules	Quantitative and Qualitative	Students	81	Gnowbe platform and classroom observation	The results showed that adult learners were keen to learn from bite-sized learning activities during these pockets of down time.
[7]	Norway	Public sector organization	To investigate the role of microlearning on cost-efficiency on knowledge transfer in a project-based organization; reflects on the design process of the lessons	Action research	Employees	334	Questionnaire	Microlearning is relevant and reduces arbitrary knowledge transfer among project teams, facilitating knowledge sharing in project-based organizations. This indicates that the short lessons were of interest to the participants. This is also confirmed by the fact that 91 % of the participants responded that the microlearning series was relevant to them. The reception was good.
[43]	–	Medical Education	Faculty development with a snippet workshop through microlearning	Quantitative and qualitative	Physician and non-physician educator	132 out of 310	Questionnaire, narrative feedback	Ninety-four percent of participants acknowledged the positive impact of snippets on educational practices, deeming the session a valuable use of their time. A concise professional development train-the-trainer workshop of snippet learning effectively facilitates the creation and utilization of this approach as well as expressing interest in a snippet repository.
[19]	Malaysia	Pharmacy	Perceptions and performance learning new knowledge through ML	Qualitative	Students	116	YouTube video analytics, quiz assessment, and online questionnaire	Online microlearning delivered knowledge equivalent to face-to-face active learning. YouTube videos and Google Forms enabled real-time monitoring and intervention. The virtual microlearning design improved student knowledge and skills using 3D molecular models and quizzes. Microlearning grants convenient access to new knowledge, while its structured format facilitates flexible learning for distance learners. The virtual micro-learning design is practical, economical, and requires minimal technological investment or training, offering easy access in open distance learning environments, especially post-COVID.
[46]	USA	Nursing	To determine the effectiveness of short wellness interventions (microlearning) in nursing courses on reducing stress and anxiety and enhancing healthy lifestyle beliefs	Quantitative	Students	142	Questionnaires	Integrating wellness interventions into nursing curricula is feasible and well-received, showing a positive trend in decreasing anxiety and stress while enhancing health and lifestyle behaviours (HLB).
[41]	USA	Journalism	The design and effects of mobile micro-course on learning efficacy and learner experience	Qualitative and quantitative	Users	35	Pre-course survey and post-course survey/pe and post-test, Semi-structured questionnaire	Mobile microlearning led to improved knowledge, increased skill confidence, and greater decision-making certainty. Opportunities for improvement lie in automating feedback, implementing timed gamified exercises, and incorporating interactive real-world content.
[40]	USA	Medical Education/Internal Medicine	Assess the impact of ML on learners' knowledge acquisition and their attitudes	Quantitative and Qualitative	Postgraduates	106	Cross-sectional Survey, case-based teaching control pre-and post-test	Most participants reported bite-size training (BST) was the best conference type. Important elements of BST were found to be distilled content, multiple short talk formats, and peer teaching. Participants taught via BST scored higher compared to case-based teaching in the immediate post-test.
[36]	Hong Kong	Post-secondary Educational Institutions	RQ1: How was ML implemented in the selected institutions? RQ2: How did the introduction of ML impact T&L in the selected institutions?	Quantitative and Qualitative	Students & teachers	496	Post-course evaluation questionnaire and teacher reflections; researchers' observation and unstructured interview follow-up	Learning is presented through small bursts of training materials that can comprehended in a short time with regard to their preferred time and location. Help institutions to survive and may provide post-pandemic improved teaching and learning experiences. The current implementation is faced with practical and pedagogical challenges. 90 satisfaction
[60]	Saudi Arabia	Clinical training	To investigate perceptions of residents on the microlearning environment in postgraduate clinical training.	Quantitative	Postgraduates	129	Questionnaire	The majority of residents had positive perceptions and were satisfied of the microlearning environment in postgraduate clinical training. Female residents and those aged 25–30 showed higher satisfaction relative to males.
[52]	France, German, Italy, Spain, UK, USA	Continuing medical education (CME)	Can microlearning modules increase engagement in continuing medical education (CME) and, consequently, improve learning outcomes?	Quantitative	Students	–	Evaluation form, pre-and post-assessment	Slight improvement in the completion of evaluation activities associated with ML program. ML is associated with an increase in participation/engagement. Microlearning demonstrates a remarkable 100-fold increase in participation compared to traditional eLearning, enabling broader reach.
[51]	South Africa	Retail bank	To determine the effectiveness of ML	Quantitative	Employees	7673	A questionnaire, standardized test and a correlation test.	Learners responded positively to microlearning (ML), with an 80 % positive rating on its effectiveness. The knowledge gained through ML led to improved work performance and business metrics. However, microlearning fell short of fully embracing the social aspect of learning.
[18]	Iran	English Institute	ML-based flipped classroom effect on students' self-regulation	Qualitative and quantitative	Students	26	Questionnaires and focus group interview	Flipping a microlearning-based EFL classroom significantly improved learners' self-regulation compared to traditional classroom instruction.
[20]	Canada and Portugal	Ophthalmology	To improve CPD learning outcome	Quantitative	Educators	Phase 1 (125), phase 2 (182)	Survey/questionnaire	The majority of the participants found MLE clear and useful. Effective in communicating clearer message of what they intend their learners to learn.
[61]	From 137 countries	Industry, national pharmacovigilance centres, and academia	Microlearning based module evaluation	Quantitative	Healthcare professionals/learners	Modules 1–4 (550); module 5 (223) including completed and partly completed responses	Two surveys/questionnaires	Microlearning-based modules developed by Uppsala Monitoring Centre (UMC) were effective in improving the knowledge and skills of healthcare professionals in pharmacovigilance. The participants were highly satisfied with the microlearning modules and nearly all would recommend them to their colleagues or others.

Note. Dash (−): cannot determine.

**Figure:**
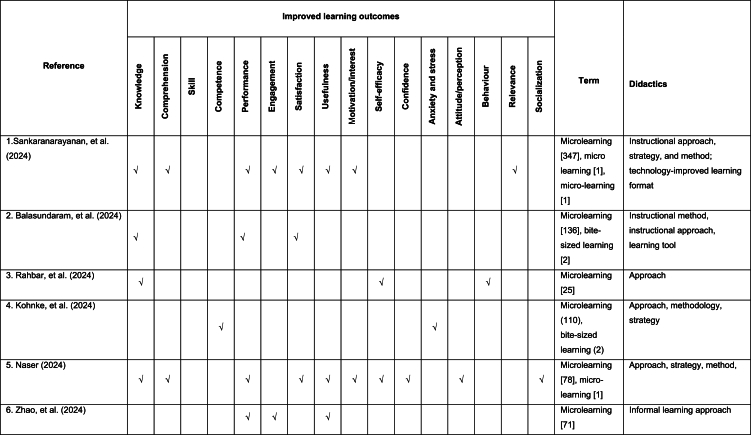

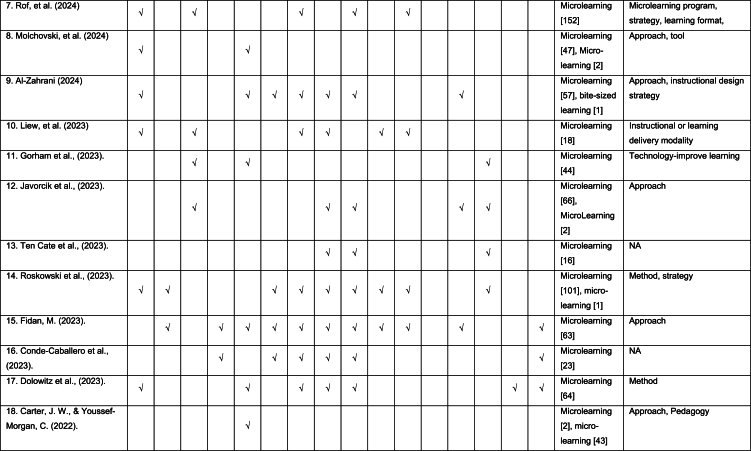

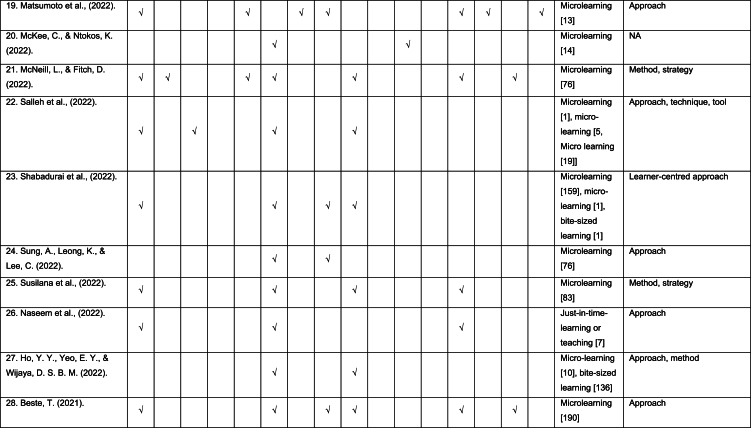

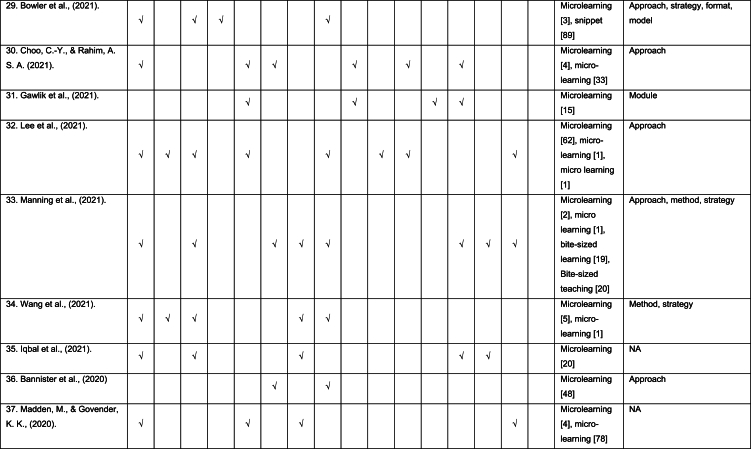

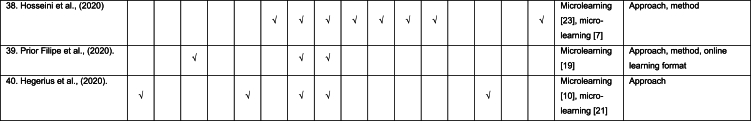


## References

[1] Qazi A., Hasan N., Owusu-Ansah C.M., Hardaker G., Dey S.K., Haruna K. (2023/01/01/2023). SentiTAM: sentiments centered integrated framework for mobile learning adaptability in higher education. Heliyon.

[2] Qazi A., Qazi J., Naseer K., Hasan N., Hardaker G., Bao D. (2024). M-Learning in education during COVID-19: a systematic review of sentiment, challenges, and opportunities. Heliyon.

[3] Qazi A. (2021). Adaption of distance learning to continue the academic year amid COVID-19 lockdown. Child. Youth Serv. Rev..

[4] Monib W.K., Qazi A., Apong R.A., Mahmud M.M. (2024). Investigating learners' perceptions of microlearning: factors influencing learning outcomes. IEEE Access.

[5] Leong K., Sung A., Au D., Blanchard C. (2021). A review of the trend of microlearning. Journal of Work-Applied Management.

[6] Robles H., Jimeno M., Villalba K., Mardini I., Viloria-Nuñez C., Florian W. (2023). Design of a micro-learning framework and mobile application using design-based research. PeerJ Computer Science.

[7] Beste T. (2021). Knowledge transfer in a project-based organization through microlearning on cost-efficiency. J. Appl. Behav. Sci..

[8] Taylor A.D., Hung W. (2022). The effects of microlearning: a scoping review. Educ. Technol. Res. Dev..

[9] Hug T., Hug T., Lindner M., Bruck a.P.A. (2005). Microlearning: Emerging Concepts, Practices and Technologies after E-Learning: Proceedings of Microlearning Conference 2005: Learning & Working in New Media.

[10] Khong H.K., Kabilan M.K. (2022). A theoretical model of micro-learning for second language instruction. Comput. Assist. Lang. Learn..

[11] Sankaranarayanan R., Leung J., Abramenka-Lachheb V., Seo G., Lachheb A. (2023). Microlearning in diverse contexts: a bibliometric analysis. TechTrends.

[12] Sung A., Leong K., Lee C. (2022). A study of learners' interactive preference on multimedia microlearning. Journal of Work-Applied Management.

[13] Zarshenas L., Mehrabi M., karamdar L., Keshavarzi M.H., keshtkaran Z. (2022). The effect of micro-learning on learning and self-efficacy of nursing students: an interventional study. BMC Med. Educ..

[14] Tabares M.S., Vallejo P., Montoya A., Correa D. (2022). A feedback model applied in a ubiquitous microlearning environment using SECA rules. J. Comput. High Educ..

[15] Zhao L., Li S., Su Y.S. (2024). Exploring college students' reading effectiveness for different types of micro-reading activities. Educ. Inf. Technol..

[16] Kohnke L., Foung D., Zou D., Jiang M. (2024). Creating the conditions for professional digital competence through microlearning. Educ. Technol. Soc..

[17] Ho Y.Y., Yeo E.Y., Wijaya D.S.B.M. (2022). Turning coffee time into teaching moments through bite-sized learning for adult learners. J. Cont. High Educ..

[18] Hosseini H.M., Ejtehadi A., Hosseini M.M. (2020). Flipping microlearning-based EFL classroom to enhance learners' self-regulation. Language Teaching Research Quarterly.

[19] Choo C.Y., Rahim A.S.A. (2021). Pharmacy students' perceptions and performance from a microlearning-based virtual practical on the elucidation of absolute configuration of drugs. Asian Journal of University Education.

[20] Prior Filipe H., Paton M., Tipping J., Schneeweiss S., Mack H.G. (2020). Microlearning to improve CPD learning objectives. Clin. Teach..

[21] Dolowitz A., Collier J., Hayes A., Kumsal C. (2023). Iterative design and integration of a microlearning mobile app for performance improvement and support for NATO employees. TechTrends.

[22] Salleh D., Khairudin N., Ibrahim M. (2022). Micro learning: motivating students' learning interests. Jurnal Psikologi Malaysia.

[23] Shabadurai Y., Chua F.F., Lim T.Y. (2022). Investigating the employees‘ perspectives and experiences of microlearning content design for online training. International Journal of Information and Education Technology.

[24] W. K. Monib, A. Qazi, and R. A. Apong, "Mapping microlearning development and trends across diverse contexts: a bibliometric analysis (2007–2023)," Interact. Learn. Environ., pp. 1-46, doi: 10.1080/10494820.2024.2402556.

[25] Prasittichok P., Smithsarakarn P. (2024). The effects of microlearning on EFL students’ English speaking: a systematic review and meta-analysis. International Journal of Learning, Teaching and Educational Research, Review.

[26] Wang C., Bakhet M., Roberts D., Gnani S., El-Osta A. (2020). The efficacy of microlearning in improving self-care capability: a systematic review of the literature. Public Health, Review.

[27] Zhang J., West R.E. (2020). Designing microlearning instruction for ProfessionalDevelopment through a competency based approach. TechTrends.

[28] Paas F., Renkl A., Sweller J. (2004/01/01 2004). Cognitive load theory: instructional implications of the interaction between information structures and cognitive architecture. Instr. Sci..

[29] Sweller J., van Merrienboer J.J.G., Paas F.G.W.C. (1998/09/01 1998). Cognitive architecture and instructional design. Educ. Psychol. Rev..

[30] Paas F.G.W.C., Van Merriënboer J.J.G. (1994/12/01 1994). Instructional control of cognitive load in the training of complex cognitive tasks. Educ. Psychol. Rev..

[31] Wong A., Leahy W., Marcus N., Sweller J. (2012/12/01/2012). Cognitive load theory, the transient information effect and e-learning. Learn. InStruct..

[32] Paas F., Tuovinen J.E., van Merriënboer J.J.G., Aubteen Darabi A. (2005/09/01 2005). A motivational perspective on the relation between mental effort and performance: optimizing learner involvement in instruction. Educ. Technol. Res. Dev..

[33] Holton E.F., Swanson R.A., Naquin S.S. (2001). Andragogy in practice: clarifying the andragogical model of adult learning. Perform. Improv. Q..

[34] Bloom B.S., Engelhart M.D., Furst E.J., Hill W.H., Krathwohl D.R.A. (1956). Handbook 1: Cognitive Domain.

[35] Dybå T., Dingsøyr T. (2008/08/01/2008). Empirical studies of agile software development: a systematic review. Inf. Software Technol..

[36] Wang T., Towey D., Ng R.Y.K., Gill A.S. (2021). Towards post-pandemic transformative teaching and learning: case studies of microlearning implementations in two post-secondary educational institutions. SN Computer Science.

[37] Fidan M. (2023). The effects of microlearning-supported flipped classroom on pre-service teachers' learning performance, motivation and engagement. Educ. Inf. Technol..

[38] Javorcik T., Kostolanyova K., Havlaskova T. (2023). Microlearning in the education of future teachers: monitoring and evaluating students' activity in a microlearning course. Electron. J. e Learn..

[39] Naseem A., Nizamuddin S., Ghias K. (2022/09/13 2022). The outcomes of a mobile just-in-time-learning intervention for teaching bioethics in Pakistan. BMC Med. Educ..

[40] Manning K.D., Spicer J.O., Golub L., Akbashev M., Klein R. (2021). The micro revolution: effect of Bite-Sized Teaching (BST) on learner engagement and learning in postgraduate medical education. BMC Med. Educ..

[41] Lee Y.M., Jahnke I., Austin L. (2021). Mobile microlearning design and effects on learning efficacy and learner experience. Educ. Technol. Res. Dev..

[42] McNeill L., Fitch D. (2022). Microlearning through the lens of gagne's nine events of instruction: a qualitative study. TechTrends.

[43] Bowler C., Foshee C., Haggar F., Simpson D., Schroedl C., Billings H. (2021). Got 15? Try faculty development on the fly: a snippets workshop for microlearning. MedEdPORTAL : the journal of teaching and learning resources.

[44] Susilana R., Dewi L., Rullyana G., Hadiapurwa A., Khaerunnisa N. (2022). Can microlearning strategy assist students' online learning?. Cakrawala Pendidikan.

[45] McKee C., Ntokos K. (2022). Online microlearning and student engagement in computer games higher education. Res. Learn. Technol..

[46] Gawlik K., Guo J., Tan A., Overcash J. (2021). Incorporating a microlearning wellness intervention into nursing student curricula. Nurse Educat..

[47] Carter J.W., Youssef-Morgan C. (2022). Psychological capital development effectiveness of face-to-face, online, and Micro-learning interventions. Educ. Inf. Technol..

[48] Roskowski S.M., Wolcott M.D., Persky A.M., Rhoney D.H., Williams C.R. (2023). Assessing the use of microlearning for preceptor development. Pharmacy.

[49] Conde-Caballero D., Castillo-Sarmiento C.A., Ballesteros-Yánez I., Rivero-Jiménez B., Mariano-Juárez L. (2023). Microlearning through TikTok in higher education. An evaluation of uses and potentials. Educ. Inf. Technol..

[50] Rahbar S., Zarifsanaiey N., Mehrabi M. (2024). The effectiveness of social media-based microlearning in improving knowledge, self-efficacy, and self-care behaviors among adult patients with type 2 diabetes: an educational intervention. BMC Endocr. Disord..

[51] Madden M., Govender K.K. (2020). The effectiveness of micro-learning in retail banking. S. Afr. J. High Educ..

[52] Bannister J., Neve M., Kolanko C. (2020). Increased educational reach through a microlearning approach: can higher participation translate to improved outcomes?. Journal of European CME.

[53] Gorham T., Majumdar R., Ogata H. (2023). Analyzing learner profiles in a microlearning app for training language learning peer feedback skills. Journal of Computers in Education.

[54] Chisega-Negrila A.-M. (2022). Microlearning for profesional development. Journal of Defense Resources Management.

[55] Nowak G., Speed O., Vuk J. (2023/01/01/2023). Microlearning activities improve student comprehension of difficult concepts and performance in a biochemistry course. Currents in Pharmacy Teaching and Learning.

[56] ten Cate D., Dikken J., Ettema R.G.A., Schoonhoven L., Schuurmans M.J. (2023). Development of a microlearning intervention regarding nursing nutritional care for older adults: a multi-methods study. Nurse Educ. Today.

[57] Scaturo D.J., Seel N.M. (2012). Encyclopedia of the Sciences of Learning.

[58] Gogus A., Seel N.M. (2012). Encyclopedia of the Sciences of Learning.

[59] Matsumoto H., Igarashi A., Hagiwara Y., Yamamoto-Mitani N. (2022). Relational Design for Dementia and Job Significance (ReDeSign): study protocol for a randomized controlled trial of an online dementia training for retail workers. Contemporary Clinical Trials Communications.

[60] Iqbal M.Z., Alaskar M., Alahmadi Y., Alhwiesh B.A., Mahrous A.A. (2021).

[61] Hegerius A., Caduff-Janosa P., Savage R., Ellenius J. (2020). E-learning in pharmacovigilance: an evaluation of microlearning-based modules developed by uppsala monitoring centre. Drug Saf..

[62] Kohnke L., Foung D., Zou D. (2024). Microlearning: a new normal for flexible teacher professional development in online and blended learning. Educ. Inf. Technol..

[63] Al-Zahrani A.M. (2024). Enhancing postgraduate students' learning outcomes through Flipped Mobile-Based Microlearning. Res. Learn. Technol..

[64] Wei X., Saab N., Admiraal W. (2021/04/01/2021). Assessment of cognitive, behavioral, and affective learning outcomes in massive open online courses: a systematic literature review. Comput. Educ..

[65] Rof A., Bikfalvi A., Marques P. (2024). Exploring learner satisfaction and the effectiveness of microlearning in higher education. Internet High Educ..

[66] Nikkhoo I., Ahmadi Z., Akbari M., Imannezhad S., Anvari Ardekani S., Lashgari H. (2023). Microlearning for today's students: a rapid review of essentials and considerations. Medical Education Bulletin.

[67] Gerbaudo R., Gaspar R., Gonçalves Lins R. (2021). Novel online video model for learning information technology based on micro learning and multimedia micro content. Educ. Inf. Technol..

[68] Zulueta L., Panoy J.F. (2022). Scenario-based microlearning strategy for improved basic science process skills in self-directed learning. International Journal of Science, Technology, Engineering and Mathematics.

[69] Hesse A., Ospina P., Wieland M., Yepes F.A.L., Nguyen B., Heuwieser W. (2019). Short communication: microlearning courses are effective at increasing the feelings of confidence and accuracy in the work of dairy personnel. J. Dairy Sci..

[70] De Gagne J.C., Woodward A., Park H.K., Sun H., Yamane S.S. (2019). Microlearning in health professions education: a scoping review protocol. JBI Database of Systematic Reviews and Implementation Reports, Review.

[71] Sankaranarayanan R., Yang M., Kwon K. (2024). Exploring the role of a microlearning instructional approach in an introductory database programming course: an exploratory case study. J. Comput. High Educ..

[72] Balasundaram S., Mathew J., Nair S. (2024). Microlearning and learning performance in higher education: a post-test control group study. Journal of Learning for Development.

[73] Molchovski P., Tokmakova K., Tokmakov D. (2024). Effectiveness of microlearning as an additional teaching instrument in orthopedics and traumatology university course. International journal of online and biomedical engineering.

[74] Liew S.C. (2023). Microlearning and online simulation-based virtual consultation training module for the undergraduate medical curriculum – a preliminary evaluation. BMC Med. Educ..

[75] Naser K.M. (2024). Tech-enhanced learning: assessing the impact of an innovative microlearning module on postgraduate students' perceptions and academic progress. International Journal of Interactive Mobile Technologies.

